# Supplementary material for: Validation of Submaximal Step Tests and the 6-Min Walk Test for Predicting Maximal Oxygen Consumption in Young and Healthy Participants
Source: Int J Environ Res Public Health. 2019 Dec 3;16(23):4858. doi: 10.3390/ijerph16234858 (PMC6926792; doi:10.3390/ijerph16234858)

Table S1. Correlation between the measured VO_{2max}, anthropometrics, and variables acquired from the submaximal tests

	VO_{2max}	Sex	Age	Weight	Height	BMI	WC	Resting HR
VO_{2max} ($mL \cdot kg^{-1} \cdot min^{-1}$)	-							
Anthropometrics								
Sex	-0.641**	-						
Age	-0.607**	0.156	-					
Weight	0.201	-0.614**	-0.017	-				
Height	0.594**	-0.748**	-0.391**	0.725**	-			
BMI	-0.276*	-0.154	0.378**	0.725**	0.114	-		
WC	-0.209	-0.336**	0.366**	0.820**	0.300*	0.854**	-	
$3MST_{20}$								
HR recovery at 30 s	-0.581**	0.527**	0.231	-0.097	-0.400**	0.212	0.179	0.758**
Average HR at 30 and 60 s	-0.564**	0.503**	0.275*	-0.114	-0.417**	0.203	0.170	0.813**
HR recovery at 60 s	-0.522**	0.457**	0.311*	-0.126	-0.417**	0.186	0.153	0.839**
$3MST_{30}$								
HR recovery at 30 s	-0.682**	0.549**	0.441**	-0.197	-0.564**	0.252*	0.124	0.671**
Average HR at 30 and 60 s	-0.673**	0.542**	0.446**	-0.212	-0.575**	0.239*	0.118	0.703**
HR recovery at 60 s	-0.648**	0.521**	0.439**	-0.223	-0.572**	0.220	0.108	0.719**
Six-minute walk test								
Distance walked (m)	0.671**	-0.506**	-0.513**	0.259*	0.567**	-0.142	-0.116	-0.308*

VO_{2max}, maximal oxygen consumption; BMI, body mass index; WC, waist circumference; HR, heart rate; $3MST_{20}$, 3-min step test using 20.3-cm step box height; $3MST_{30}$, 3-min step test using 30-cm step box height. * $p < 0.05$; ** $P < 0.01$.

Table S2. Correlations between the measured VO_{2max} and model-predicted VO_{2max} in the 3-min step test using a 20-cm step height. The prediction equation model was developed using randomly selected two-third of the participants, and their value was compared with that of the non-selected one-third of the participants. The same methods were repeated three times.

A. Model Building Set = Group 1 and 2 (N = 44), Validation Set = Group 3 (N = 22)

	β	SE	t	p
Constant	89.60	6.29	14.24	0.00
Gender	-11.37	2.12	-5.38	0.00
Age	-0.49	0.09	-5.30	0.00
Weight	-0.19	0.07	-2.96	0.01
HRR 30s	-0.08	0.06	-1.25	0.22
	r	Adj. R-square	Predicted VO_{2max}	SEE
	0.87	0.73	37.10 (8.10)	4.80

B. Model Building Set = Group 1 and 2 (N = 44), Validation Set = Group 3 (N = 22)

	β	SE	t	p
Constant	85.29	6.70	12.72	0.00
Gender	-11.18	1.88	-5.95	0.00
Age	-0.42	0.07	-6.18	0.00
Weight	-0.13	0.06	-2.13	0.04
HRR 30s	-0.09	0.05	-1.88	0.07
	r	Adj. R-square	Predicted VO_{2max}	SEE
	0.89	0.773	37.68 (7.79)	4.21

C. Model Building Set = Group 2 and 3 (N = 44), Validation Set = Group 1 (N = 22)

	β	SE	t	p
Constant	82.13	7.34	11.19	0.00
Gender	-9.93	2.68	-3.71	0.00
Age	-0.43	0.08	-5.51	0.00
Weight	-0.07	0.08	-0.94	0.35
HRR 30s	-0.13	0.07	-1.97	0.06
	r	Adj. R-square	Predicted VO_{2max}	SEE
	0.85	0.702	37.22 (7.84)	5.01

All participants were randomly divided into three groups (1, 2, or 3). Then data from two of three groups were used to develop prediction equation for VO_{2max} value. This equation was used to validate predicted VO_{2max} value with measured VO_{2max}. Data were analyzed with linear regression. HRR 30s, heart rate recovery at 30 s after cessation.

Table S3. Correlations between the measured VO_{2max} and model-predicted $\dot{V}O_{2max}$ in the 3-min step test using a 30-cm step height. The prediction equation model was developed using randomly selected two-third of the participants, and their value was compared with that of the non-selected one-third of the participants. The same methods were repeated three times.

A. Model Building Set = Group 1 and 2 (N = 49), Validation Set = Group 3 (N = 24)				
	β	SE	t	p
Constant	86.74	7.27	11.94	0.00
Gender	-10.71	2.37	-4.52	0.00
Age	-0.44	0.10	-4.50	0.00
Weight	-0.17	0.07	-2.29	0.03
HRR 30s	-0.07	0.05	-1.44	0.16
	r	Adj. R-square	Predicted $\dot{V}O_{2max}$	SEE
	0.83	0.66	37.89 (7.54)	5.35

B. Model Building Set = Group 1 and 2 (N = 48), Validation Set = Group 3 (N = 25)				
	β	SE	t	p
Constant	80.24	6.94	11.55	0.00
Gender	-8.63	2.02	-4.28	0.00
Age	-0.42	0.09	-4.69	0.00
Weight	-0.10	0.07	-1.50	0.14
HRR 30s	-0.09	0.05	-2.02	0.05
	r	Adj. R-square	Predicted $\dot{V}O_{2max}$	SEE
	0.85	0.69	37.30 (6.91)	4.57

C. Model Building Set = Group 2 and 3 (N = 49), Validation Set = Group 1 (N = 24)				
	β	SE	t	p
Constant	85.38	5.28	16.18	0.00
Gender	-11.48	1.78	-6.45	0.00
Age	-0.37	0.06	-5.80	0.00
Weight	-0.12	0.05	-2.45	0.02
HRR 30s	-0.10	0.04	-2.66	0.01
	r	Adj. R-square	Predicted $\dot{V}O_{2max}$	SEE
	0.91	0.80	37.34 (8.31)	4.04

All participants were randomly divided into three groups (1, 2, or 3). Then data from two of three groups were used to develop prediction equation for VO_{2max} value. This equation was used to validate predicted VO_{2max} value with measured VO_{2max}. Data were analyzed with linear regression. HRR 30s, heart rate recovery at 30 s after cessation.

Table S4. Correlations between the measured VO_{2max} and model-predicted VO_{2max} in the 6-minute walk test. The prediction equation model was developed using randomly selected two-third of the participants, and their value was compared with that of the non-selected one-third of the participants. The same methods were repeated three times.

A. Model Building Set = Group 1 and 2 (N = 43), Validation Set = Group 3 (N = 21)				
	β	SE	t	p
Constant	58.54	10.18	5.75	0.00
Gender	-12.42	1.74	-7.16	0.00
Age	-0.32	0.07	-4.46	0.00
Weight	-0.18	0.05	-3.48	0.00
SMWD/10	0.27	0.09	2.92	0.01
	r	Adj. R-square	Predicted VO_{2max}	SEE
	0.91	0.82	36.97 (8.45)	3.95

B. Model Building Set = Group 1 and 2 (N = 43), Validation Set = Group 3 (N = 21)				
	β	SE	t	p
Constant	60.61	12.07	5.02	0.00
Gender	-10.53	2.05	-5.14	0.00
Age	-0.37	0.10	-3.86	0.00
Weight	-0.17	0.07	-2.44	0.02
SMWD/10	0.21	0.11	1.94	0.06
	r	Adj. R-square	Predicted VO_{2max}	SEE
	0.85	0.7	37.30 (6.91)	4.94

C. Model Building Set = Group 2 and 3 (N = 42), Validation Set = Group 1 (N = 22)				
	β	SE	t	p
Constant	64.12	12.60	5.09	0.00
Gender	-10.49	2.19	-4.80	0.00
Age	-0.43	0.10	-4.15	0.00
Weight	-0.17	0.07	-2.51	0.02
SMWD/10	0.18	0.10	1.82	0.08
	r	Adj. R-square	Predicted VO_{2max}	SEE
	0.855	0.7	36.23 (7.77)	4.99

All participants were randomly divided into three groups (1, 2, or 3). Then data from two of three groups were used to develop prediction equation for VO_{2max} value. This equation was used to validate predicted VO_{2max} value with measured VO_{2max}. Data were analyzed with linear regression. SMWD, six-minute walked distance.

Figure S1. Correlations between the measured VO_{2max} and model-predicted VO_{2max} in the 3-min step test using a 20-cm step height. The prediction equation model was developed using randomly selected two-third of the participants, and their value was compared with that of the non-selected one-third of the participants. The same methods were repeated three times.

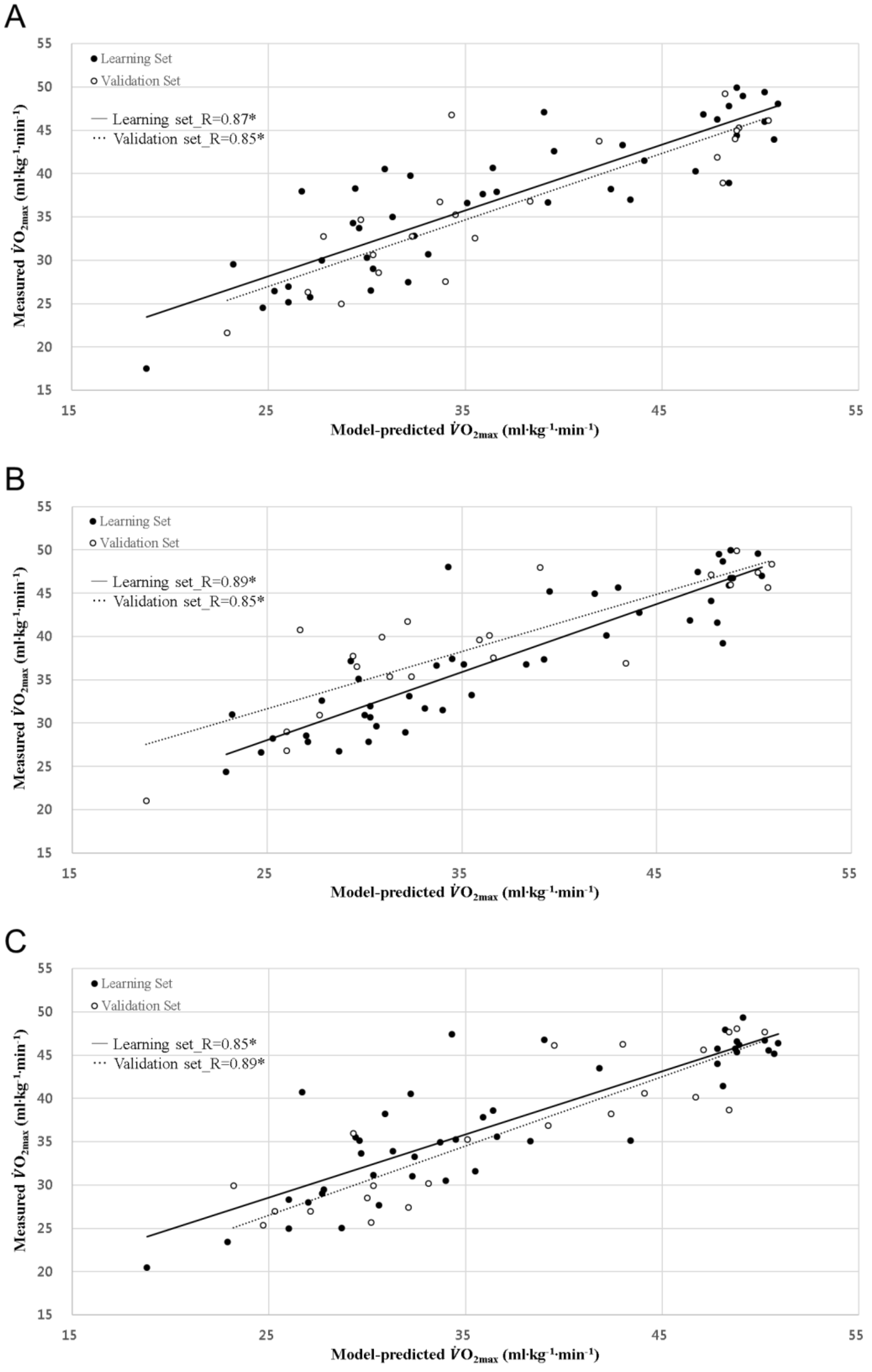

Figure S2. Correlations between the measured VO_{2max} and model-predicted VO_{2max} in the 3-min step test using a 30-cm step height. The prediction equation model was developed using randomly selected two-third of the participants, and their value was compared with that of the non-selected one-third of the participants. The same methods were repeated three times.

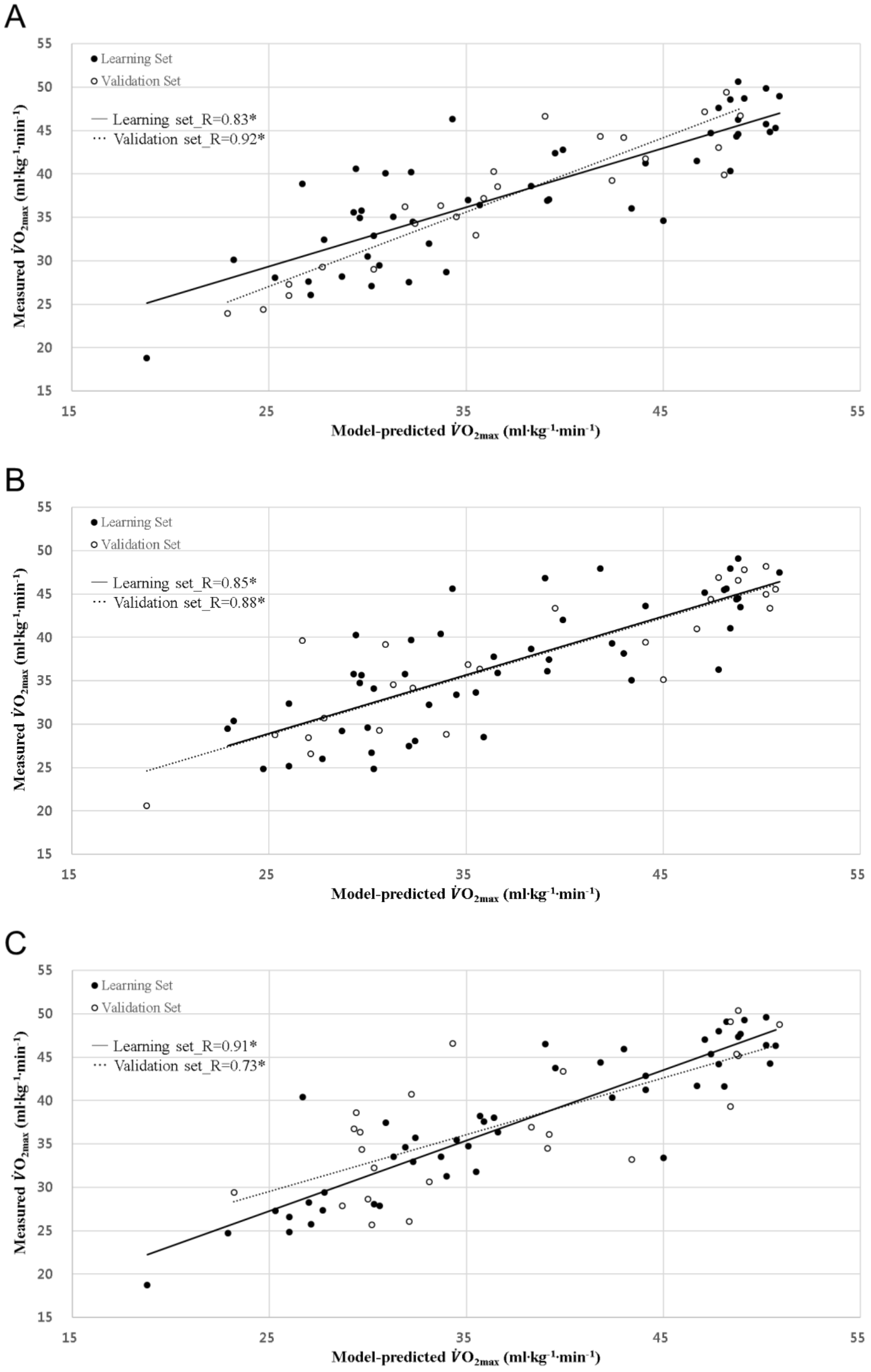

Figure S3. Correlations between the measured VO_{2max} and model-predicted VO_{2max} in the 6-minute walk test. The prediction equation model was developed using randomly selected two-third of the participants, and their value was compared with that of the non-selected one-third of the participants. The same methods were repeated three times.

A

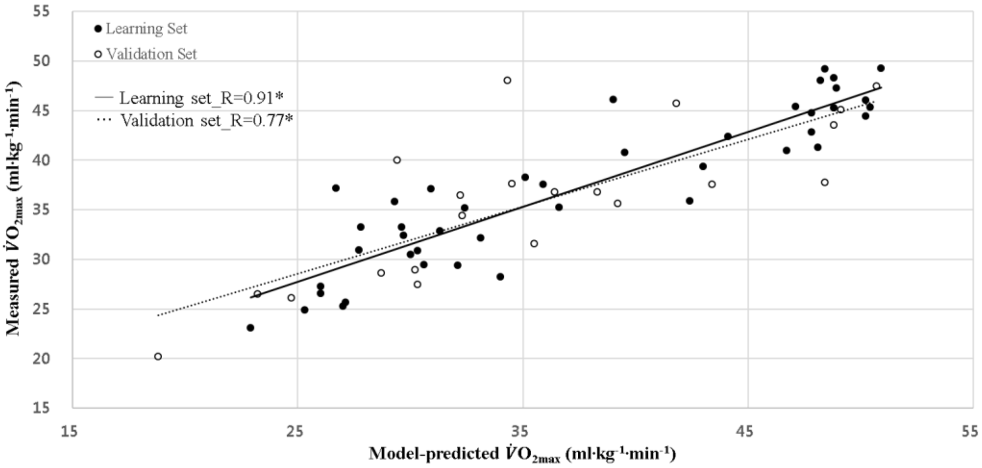

B

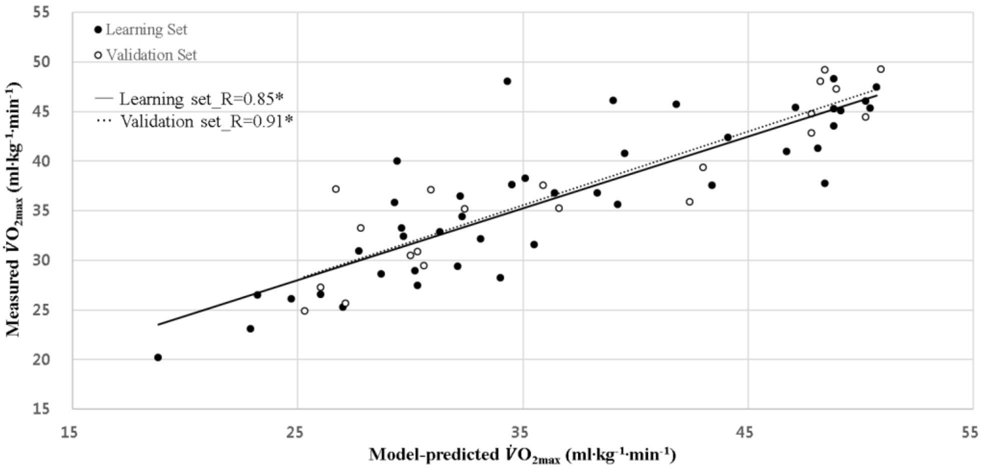

C

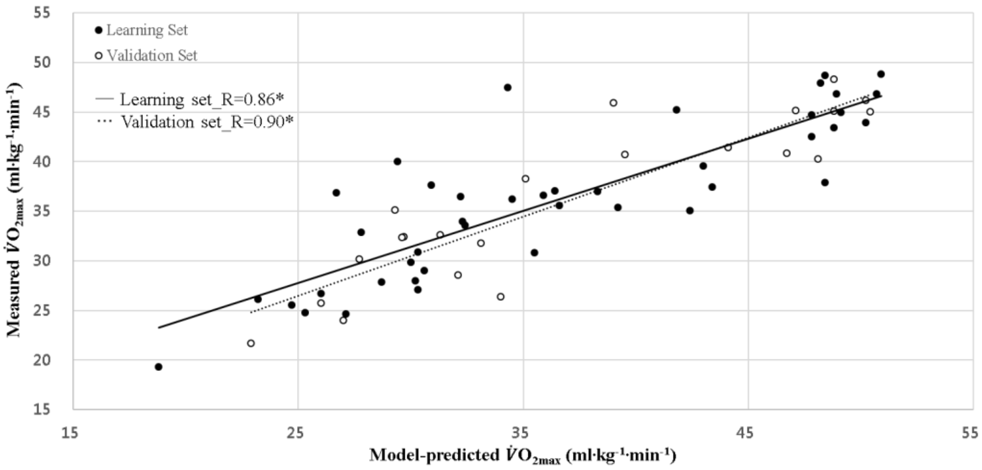

Supplement: Supplementary file 1 [file ijerph-16-04858-s001.pdf]
